# Does cannabidiol reduce the adverse effects of cannabis in schizophrenia? A randomised, double-blind, cross-over trial

**DOI:** 10.1038/s41386-025-02175-3

**Published:** 2025-07-24

**Authors:** Edward Chesney, Dominic Oliver, Ananya Sarma, Ayşe Doğa Lamper, Ikram Slimani, Millie Lloyd, Alex M. Dickens, Michael Welds, Matilda Kråkström, Irma Gasparini-Andre, Matej Orešič, Will Lawn, Natavan Babayeva, Tom P. Freeman, Amir Englund, John Strang, Philip McGuire

**Affiliations:** 1 Department of Addictions, Institute of Psychiatry, Psychology and Neuroscience, King’s College London, London, UK; 2 Department of Psychosis Studies, Institute of Psychiatry, Psychology and Neuroscience, King’s College London, London, UK; 3 South London and Maudsley NHS Foundation Trust, London, UK; 4 Department of Psychiatry, University of Oxford, Warneford Hospital, Oxford, UK; 5 NIHR Oxford Health Biomedical Research Centre, Oxford, UK; 6 Turku Bioscience Centre, University of Turku and Åbo Akademi University, Turku, Finland; 7 Department of Life Technologies, University of Turku, Turku, Finland; 8 School of Medical Sciences, Örebro University, Örebro, Sweden; 9 Department of Psychology, Institute of Psychiatry Psychology and Neuroscience, King’s College London, London, UK; 10 Addiction and Mental Health Group (AIM), Department of Psychology, University of Bath, Bath, UK

**Keywords:** Experimental models of disease, Human behaviour, Psychosis

## Abstract

In patients with schizophrenia, cannabis use exacerbates symptoms and can lead to a relapse of psychosis. Some experimental studies in healthy volunteers suggest that pre-treatment with cannabidiol (CBD) may reduce these effects, but others do not. Here, we investigated whether pre-treatment with CBD ameliorates the acute adverse effects of cannabis in patients with schizophrenia. Participants (*n* = 30) had schizophrenia or schizoaffective disorder plus a comorbid cannabis use disorder. In a double-blind, randomised, placebo-controlled, crossover trial, participants received oral CBD 1000 mg or placebo three hours before inhaling vaporised cannabis (containing Δ^9^-tetrahydrocannabinol (THC) 20–60 mg). The primary outcome was delayed verbal recall measured with the Hopkins Verbal Learning Test-Revised. We also measured psychotic symptoms with the Positive and Negative Syndrome Scale (PANSS) – positive subscale. Delayed verbal recall after cannabis administration was 3.5 words (95% confidence interval [CI]: 2.5–4.5) following pre-treatment with CBD, compared to 4.8 words (95% CI: 3.9 to 5.8) following pre-treatment with placebo (mean difference [MD] = −1.3 [95% CI: −2.0 to −0.6]; *p* = 0.001). After CBD pre-treatment, inhalation of cannabis was associated with an increase in PANSS-P score of 5.0 (95% CI: 3.6 to 6.5), compared to 2.9 (95% CI: 1.5 to 4.3) following pre-treatment with placebo (MD = 2.2 [95% CI: 0.6 to 3.7]; *p* = 0.01). Administration of CBD did not have a significant effect on plasma concentration of THC or its active metabolite, 11-hydroxy-THC. In patients with schizophrenia and a comorbid cannabis use disorder, pre-treatment with CBD did not attenuate the acute effects of cannabis on memory impairment or psychotic symptoms, but appeared to exacerbate them. The study was registered on Clinicaltrials.gov (NCT04605393).

## Introduction

In people with schizophrenia, cannabis use is associated with acute exacerbations of symptoms and an increased risk of relapse [1, 2]. Many people with schizophrenia use cannabis [3], and about one in five have a comorbid cannabis use disorder (CUD) [4]. Managing CUD in people who have schizophrenia is difficult, as there are no specific interventions that are effective in this group [5, 6].

The adverse effects of cannabis are attributable to its constituent Δ^9^-tetrahydrocannabinol (THC), a partial agonist at the cannabinoid receptor 1 (CB1) [7, 8]. Preclinical data suggest that the effects of THC on memory and behaviour may be mediated by intracellular CB1 receptors which are located on the mitochondria of neurons and astroglia [9, 10]. Neuroimaging studies have suggested that the hippocampus, which is densely populated with CB1 receptors, may mediate many of the behavioural effects of cannabis [11]. When THC is given intravenously to people with schizophrenia, it transiently increases psychotic symptoms and cognitive impairments [12]. In people with both schizophrenia and cannabis use disorder, an oral dose of THC (15 mg) impaired cognition but did not exacerbate psychotic symptoms, while smoked cannabis (THC 3.5%) did not affect either outcome [13]. Another study found that smoked cannabis increased paranoia and impaired cognition in cannabis users at clinical high risk for psychosis, but not in healthy cannabis users [14]. Several controlled studies in healthy volunteers have found that inhaled THC can impair cognition and induce psychotic symptoms when compared to placebo [15, 16].

Cannabidiol (CBD) is another constituent of cannabis and has a similar molecular structure to THC. Its mechanism of action is not established, but it may act as a negative allosteric modulator of CB1 receptors, altering their response to both endogenous agonists, such as anandamide and 2-arachidonoylglycerol, and exogenous agonists, such as THC [17, 18]. Preclinical studies have also suggested that it can modulate mitochondrial function [19–22], or that its antipsychotic effects may depend on activity at 5-HT1A and TRPV1 receptors [23]. Data from clinical trials suggest that CBD (at doses between 600 and 1000 mg) can reduce psychotic symptoms in people with schizophrenia [24–26], and in people at clinical high risk of the disorder [27]. It also has a relatively benign adverse event profile and has a high level of acceptability to people with psychosis [28, 29], and is thus a promising candidate treatment [30].

Several experimental studies in healthy volunteers have investigated whether CBD can modulate the acute effects of cannabis or THC [31]. However, these have produced mixed results. Two studies reported that pre-treatment with CBD (either as a high oral dose or intravenously) attenuated the acute effects of subsequent intravenous THC on psychotic symptoms and memory impairment [32, 33]. In contrast, three studies which co-administered CBD and THC (via the oral route) reported that CBD exacerbated the effects of THC on outcomes such as cognition, anxiety, and subjective level of intoxication [34–36]. Finally, three studies that co-administered inhaled CBD and THC found that CBD had no influence on THC’s effects [15, 16, 37]. The differences between studies could be related to differences in whether CBD is given as a pre-treatment or is administered at the same time as THC, the route of administration of CBD and THC (oral/inhaled/intravenous), and the doses of CBD and THC used. For example, when CBD and THC are co-administered orally, CBD may increase the effects of THC by inhibiting hepatic first-pass metabolism [34]. If too low a dose of THC is used it may fail to induce adverse effects, precluding the detection of modulatory effects of CBD [15].

The present study was designed to assess whether pre-treatment with CBD could reduce the acute adverse effects of cannabis in people with schizophrenia. The study was a double-blind, randomised, placebo-controlled, crossover trial conducted in a Clinical Research Facility. To account for drug tolerance, participants who did not experience a significant increase in psychotic symptoms with the standard THC dose (20 mg) were invited to complete additional experiments with higher doses of THC (40 mg and then 60 mg).

We tested the hypotheses that, relative to placebo, pre-treatment with CBD (1000 mg oral) would reduce the severity of:

i. cannabis-induced impairment in delayed verbal recall.
ii. cannabis-induced positive psychotic symptoms.

## Methods and materials

### Design

A randomised, double-blind, two-arm, crossover laboratory study, conducted between October 2021 and July 2023 at the NIHR Clinical Research Facility at King’s College Hospital, London, UK.

### Participants

We recruited individuals receiving secondary mental healthcare from the South London and Maudsley NHS Foundation Trust, London, UK. The inclusion criteria were: age 18-65 years; diagnosis of schizophrenia or schizoaffective disorder (satisfying ICD-10 criteria); clinically stable for at least three months; regular (at least weekly) cannabis use for the past 3 months or more; evidence from either clinicians or from the patient that cannabis use exacerbates their symptoms or increases their risk of relapse (assessed via review of clinical notes, clinician reports, and participant self-report); treatment with regular doses of antipsychotic medication for at least 1 month; participant agrees to abstain from cannabis use for at least 24 hours prior to study visits; willing to have an intravenous cannula inserted to collect blood samples on experimental visits; sufficiently fluent in English; and providing written informed consent. Exclusion criteria were: extreme cannabis use (estimated to use over 2 grams of cannabis/day); dependence on alcohol or illicit substances other than cannabis as defined by ICD-10; pregnancy (current or planned) or breastfeeding; physical health disorder or another mental health disorder that may influence the patient’s ability to tolerate the procedure, or that may alter the results of the study; participant has taken part in any drug study within the last 3 months or taking part in another study over the course of the trial; drug sensitivity/allergy to cannabis or lorazepam; unlikely to be able to complete experiments for any reason.

### Screening visit

After providing written informed consent, participants were assessed against inclusion and exclusion criteria, completed a physical health assessment, provided a urine and breath sample for drug and alcohol screening tests, and provided a blood sample to confirm adherence to oral antipsychotic medication. Once these were completed, participants practised the study procedures, i.e. using the vaporiser and completing cognitive and psychopathological assessments.

### Randomisation and blinding

The randomisation schedule was developed by an independent statistician and shared with the dispensing pharmacy only. To maintain the balance of treatment allocation, a block randomisation was used, with a block size of four. The study was double-blind, with both participants and researchers blind to treatment allocation. The CBD and placebo capsules had identical appearance.

Participants were asked to guess treatment allocation prior to cannabis intoxication on the first experiment and at the end of the second experiment. Researchers guessed treatment allocation at the end of the second experiment only.

### Study drugs

CBD capsules and matching placebo were obtained from BSPG Laboratories, UK. The capsules contained 200 mg of naturally derived CBD which had been refined to >99.9% purity, with no detectable THC, Δ^8^-THC or cannabinol. The CBD was dissolved in Softisan 378, a medium-chain fatty acid, containing palmitoyl ascorbic acid, which is solid at room temperature.

Two cannabis-based products for medical use (CBPMs) were used during the study: Adven EMT-1 (THC: 19%; CBD < 1%) and Pedanios 20/1 (THC: 18.6–19.8%; CBD < 0.1%), provided by Rokshaw Laboratories, UK and IPS Pharma, UK, respectively.

### Drug administration and inhalation procedure

CBD 1000 mg (5 × 200 mg capsules) was administered with food (typically two yoghurts [238–296 kCal; 6.7–8.1 grams of fat]).

For the cannabis challenge, ground cannabis flower, containing THC 20 mg (four standard THC units [38]), was vaporised and then inhaled by participants. Cannabis inhalation began three hours after CBD administration (aligning with CBD’s expected peak plasma concentration [39]) and followed the method used by Englund and colleagues [37]. The cannabis was vaporised at 210 °C into a transparent polythene balloon using a Volcano® Medic Vaporiser (Storz-Bickel GmbH, Tüttlingen, Germany). Participants were instructed to hold their breath for 8 seconds after each inhalation, with an 8 second break between inhalations. After a short break (1–2 minutes), the procedure was repeated, and the cannabis was heated again to inflate a second balloon.

### Dose-escalation procedure

To take into account drug tolerance, and to increase the power of the study, participants who did not demonstrate a significant psychotic response to THC 20 mg (defined as an increase in PANSS-positive subscale of ≥3 during either experiment) [32, 37, 40] were invited to complete additional pairs of experiments at higher doses (Figure S1). The higher doses were 40 mg (eight standard THC units) and then 60 mg (12 standard THC units). Participants were re-randomised.

### Experimental procedure

At the start of each experiment, participants provided a urine sample to confirm recent abstinence from substances (apart from cannabis), completed a breath test to confirm recent abstinence from alcohol, and were judged as being sober by the study psychiatrist. Psychopathological outcome measures were completed prior to CBD/placebo administration and were then repeated at later timepoints (Figure S2). Cognitive testing was completed once during each experiment, starting 20 minutes after cannabis inhalation was completed. Caffeinated drinks and cigarette breaks were allowed ad libitum. At the end of the study visit, participants were discharged after completing a sobriety test.

### Outcome measures

#### Hopkins verbal learning test-revised (HVLT-R)

The primary outcome measure was delayed verbal recall on the HVLT-R [41]. A review of the effects of THC on cognition found that some of the largest impairments occur on tasks measuring verbal learning and memory [42]. The HVLT-R was completed at screening and once during each experiment, 20 minutes after cannabis intoxication. The researcher reads a list of 12 words to the participant and asks them to repeat aloud as many words as they can remember. This has been completed three times. The total number of words recalled across the three learning trials was the immediate recall score. After 20–25 minutes, participants are asked to recall the list of words, to determine delayed recall. An intrusion was a word which was not in the list. Repetitions referred to the number of times a correctly recalled word was repeated. A unique list of words was used on each study visit.

#### The positive and negative syndrome scale (PANSS) - positive and negative subscales

The Positive and Negative Syndrome Scale is the gold-standard assessment for measuring symptom severity in schizophrenia [43]. We assessed the Positive and Negative subscales. The Positive Subscale (PANSS-P) was the psychopathological outcome of primary interest. A semi-structured interview was conducted by a psychiatrist trained in PANSS assessment and was complemented by observations from other researchers. The PANSS is typically used to assess psychotic symptoms over the past week. In the present study, the PANSS was used to measure acute symptom changes, an approach that has been used in several previous experimental cannabis studies [12, 13, 16, 32, 37]. The initial interview assessment was completed prior to CBD/placebo administration. The post-THC assessment included an interview at 0–20 minutes after inhalation and a second interview/review at the end of the experiment.

#### The psychotomimetic states inventory (PSI)

The PSI was used to assess subjective psychotic symptoms [44]. The questionnaire includes 48 items organised into six domains: delusory thinking, perceptual distortion, cognitive disorganization, anhedonia, mania, and paranoia. The PSI was completed prior to CBD/placebo administration, and at least 90 minutes after cannabis administration. Participants were instructed to describe their experiences over the last few hours, or since receiving cannabis.

#### The state social paranoia scale (SSPS)

The SSPS is a ten-item questionnaire to assess persecutory thoughts [45]. The SSPS was completed prior to CBD/placebo administration, and at least 90 minutes after cannabis administration. Participants were instructed to describe their experiences over the last few hours, or since receiving cannabis.

#### The state-trait anxiety inventory-state scale (STAI-S)

The STAI-S is a 20-item scale for assessing anxiety symptoms [46]. The STAI-S was completed at three timepoints: prior to CBD/placebo administration, immediately prior to cannabis administration, and 20 minutes after cannabis administration.

#### Forward and reverse digit span

This measure of working memory involves the recall of increasingly long sequences of numbers in forward and reverse order. The task continues until the participant fails two consecutive attempts at a sequence. It was completed 25 minutes after cannabis administration.

#### Visual analogue scales (VAS)

These were used to measure other subjective effects. Participants marked on a 100 mm horizontal line to indicate the level of a given feeling at that moment (0 mm ‘Not at all’ to 100 mm ‘Extremely’). The feeling states included: ‘Feel drug effect’, ‘Like drug effect’, ‘Want more drug’, ‘Thinking clearly’, ‘Tired’, ‘Excited’, ‘Want to talk’, ‘Anxious’, ‘Relaxed’, ‘Happy,’ ‘Irritable’, ‘Suspicious’, ‘Hearing voices’, ‘Dry mouth’, ‘Hungry’, ‘Vulnerable’, and ‘Threatened’. VAS were complete at seven timepoints: baseline, 90 minutes post-CBD/placebo, immediately prior to cannabis administration, 10-, 45- and 90-minutes post-cannabis, and at the end of the study visit.

#### Pharmacokinetics

Blood samples were collected at six timepoints: pre-CBD/placebo administration, 90 minutes post-CBD/placebo, and at 0-, 5-, 15- and 90-minutes post-cannabis inhalation. The concentration of cannabinoids was quantified using ultra high-performance liquid chromatography-mass spectrometry.

#### Physiological outcomes

Heart rate, blood pressure and temperature were measured prior to CBD/placebo administration, 90 minutes post-CBD/placebo, at 5-, 15- and 90-minutes post-cannabis inhalation, and at discharge.

### Statistical analysis

#### Registration

The study was registered on Clinicaltrials.gov (NCT04605393) and Open Science Framework (https://osf.io/2y4n8/). The statistical analysis plan was registered prior to unblinding of treatment allocation (https://osf.io/xswdm).

#### Primary analysis population

Since this is an experimental study, which aims to understand the efficacy of CBD under ideal conditions, not its effectiveness as a clinical intervention, our primary analyses were per-protocol. For participants who completed more than one pair of experiments, only the data from the experiments where they received the highest dose of THC were included.

#### Sensitivity analyses

We completed two sensitivity analyses for delayed verbal recall and change in PANSS-P: i) with the intention to treat population (highest dose received) (*n* = 34), and ii) using data from THC 20 mg experiments only (*n* = 30).

#### Analysis of psychopathology, cognition and physiology

Continuous outcomes are reported as means with standard deviation (SD). Categorical outcomes are reported as frequencies. We used linear mixed models to assess the effect of CBD on continuous outcomes. Treatment allocation (CBD/placebo) and visit number were included as fixed effects, with participant as a random effect to account for the dependency between repeated measures. Estimated marginal means were calculated and compared in a pairwise test. Treatment effects on rate of clinically significant PANSS-P increases (≥3) were assessed with McNemar’s test.

#### Pharmacokinetic analyses

Pharmacokinetic parameters were summarized descriptively. We reported the geometric mean of the peak plasma concentration (C_max_) and plasma exposure over time (AUC_t_). AUC_t_ was calculated using the trapezoid method implemented in the bayestestR package (version 0.13.0). To compare the C_max_ and AUC_t_ of pharmacokinetic parameters we followed the same analysis strategy as for psychopathological and cognitive outcomes.

#### Imputation of data

There was no missing data for cognitive and psychopathological outcomes. Multiple imputation chain equations (MICE) were used to impute missing values in pharmacokinetic outcomes using the mice package (version 3.16.0) in R. A total of 37/360 (10%) data points were missing.

#### Order effects

There was sufficient time for drug washout between experiments (minimum 1 week), so carryover effects were considered unlikely, but due to learning and familiarisation with the study protocol and lab environment, a period effect may have occurred. We therefore included visit number in all models to account for potential order effects.

#### Correction for multiple comparisons

Benjamini-Hochberg correction was conducted across outcomes locally i.e. across secondary cognitive outcomes, secondary psychopathological outcomes, VAS, and physiological outcomes. P values below 0.05 were considered significant. All tests were two-tailed.

#### Software

All analyses were conducted using R version 4.2.2. lme4 (version 1.1-31) was used to fit the linear mixed effects models and estimated marginal mean (EMM) contrasts were calculated using the emmeans package (version 1.8.4-1).

#### Ethical and regulatory approvals

The study was approved by the Health and Social Care Research Ethics Committee A (Reference Number: 20/NI/0074). All participants provided written informed consent and the study was conducted in compliance with the principles of Good Clinical Practice, and the Declaration of Helsinki (1996).

### Results

#### Recruitment, pilot experiments and dose-escalation

273 individuals were screened for inclusion, of which 92 were invited to an assessment (Figure S3). Of these, 43 attended the assessment and 37 completed at least one experiment. Three participants completed pilot experiments with low doses of THC (10 mg and 15 mg) only, and four participants dropped out after their first experiment.

Thirty participants finished the study per-protocol, each having completed two experiments with THC 20 mg. Of these, 19 had a significant response to the cannabis challenge (PANSS-P increase ≥3). Eleven participants were eligible to repeat the experiments with THC 40 mg of which six volunteered. One participant did not respond to THC 40 mg, so they completed two experiments with THC 60 mg. Five participants did not complete higher doses due to: loss to follow-up (*n* = 2), alcohol use disorder relapse, death, and declined.

#### Reasons for withdrawal and adverse events

Four participants dropped out of the study after their first experimental visit (Figure S3). One withdrew due to a newly diagnosed medical condition, unrelated to the study procedures. Another discontinued treatment with antipsychotic medication prior to their second experiment. One participant disagreed with the timing of blood sampling and therefore withdrew.

One participant was withdrawn due to a treatment-related adverse event. During their first experiment, soon after receiving cannabis, they developed persecutory delusions related to the study team. By the following day, and without additional intervention, these symptoms resolved completely. One other participant experienced a treatment-related adverse event. After receiving the cannabis, their blood pressure reached 224/78 mm Hg. By the end of the experiment it had normalised without intervention. They did not withdraw from the study as it occurred during their second experiment. Both treatment-related adverse events occurred after treatment with CBD.

#### Per-protocol analysis

The demographic and clinical characteristics of the per-protocol study population are described in Table 1. The characteristics of the intention-to-treat and higher dose study populations are reported in Table S1. There was no difference in the time taken to complete the cannabis administration procedure between the two arms (CBD: 11.8 minutes; Placebo: 11.5 minutes; *p* = 0.76).

**Table 1:** Demographic and clinical characteristics of the per-protocol study population (*n* = 30).

	Age (years)	39.7 (11.1)
Sex	Male	28 (93%)
	Female	2 (7%)
Ethnicity	Black	17 (57%)
	Mixed/Other	7 (23%)
	White	6 (20%)
Employed	Yes	3 (10%)
	No	27 (90%)
Primary diagnosis (ICD-10 criteria)	Schizophrenia	27 (90%)
	Schizoaffective disorder	3 (10%)
Illness duration and severity	Years of illness	17.1 (11.5)
	Relapses in past 10 years	2.6 (2.5)
Treatment and management	Olanzapine equivalents (mg)	14.4 (7.7)
	Long-acting injectable antipsychotic (current)	25 (83%)
	Clozapine (current)	4 (13%)
	Community Treatment Order (ever)	7 (23%)
Baseline symptom severity	PANSS Positive subscale	13.9 (5.5)
	PANSS Negative subscale	13.3 (6.0)
	AUDIT score	4.3 (4.2)
	CUDIT score	15.9 (4.9)
Cannabis use disorder (DSM-5)	Mild	1 (3%)
	Moderate	2 (7%)
	Severe	27 (90%)
Cannabis use	Days per week	5.4 (1.9)
	Joints per day	5.3 (4.2)
	Grams per day	1.1 (0.7)
	High potency	24 (80%)
	Low potency	6 (20%)
	Tobacco in joints	30 (100%)
Tobacco use^a^	Yes	25 (83%)
	No	5 (17%)

Data are *n* (%) or mean (SD).

*DSM-5* Diagnostic and Statistical Manual of Mental Disorders, Fifth Edition, *AUDIT* Alcohol use disorders identification test, *CUDIT* Cannabis use disorder identification test.

^a^Independent of cannabis use.

**Fig. 1:**
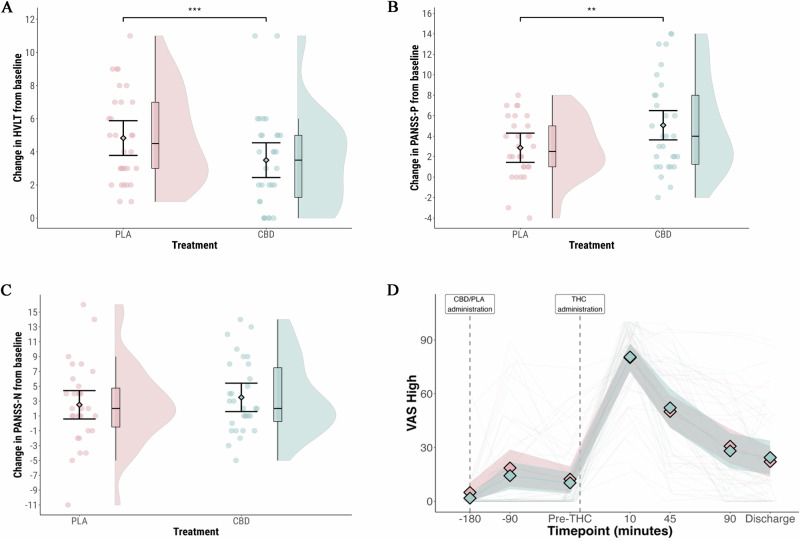
The effect of CBD treatment on core cognitive and psychopathological outcome measures. **A** Delayed verbal learning on the HVLT-R; **B** Positive psychosis symptoms measured by the PANSS-Positive Subscale; **C** Negative psychosis symptoms measured by the PANSS-Negative Subscale; **D** Participant rated level of intoxication measured with a visual analogue scale for ‘Feel Drug Effect’. Blue: CBD pre-treatment arm; Pink: Placebo pre-treatment arm. Shaded areas in violin plots (1A-C) represent distribution density. In Fig. 1D, the shaded areas represent 95% confidence intervals. **p = 0.01 ***p = 0.001.

The primary outcome was delayed verbal recall after cannabis administration (Table 2, Fig. 1A). Lower scores indicate worse performance. In the CBD arm, the mean score was 3.5 (95% CI: 2.5 to 4.5); in the placebo arm, it was 4.8 (95% CI: 3.9 to 5.8), a difference that was statistically significant (MD = -1.3 [95% CI: -2.0 to -0.6]; *p* = 0.001). There was no effect of visit (*p* > 0.05). Sensitivity analyses produced the same pattern of results (Tables S2 and S3).

**Table 2:** Cognitive outcome measures.

			CBD	Placebo	CBD vs. Placebo		
		Screening visit	Post-cannabis	Post-cannabis	Mean difference	*P* value	Corrected *P* value
HVLT-R	Immediate recall	19.3 (17.2 to 21.4)	14.7 (12.8 to 16.7)	16.6 (14.6 to 18.5)	−1.8 (−3.5 to −0.2)	0.04	0.14
	Immediate intrusions	0.9 (0.6 to 1.2)	2.1 (0.9 to 3.2)	1.9 (0.8 to 3.1)	0.1 (−0.8 to 1.0)	0.80	0.80
	Immediate repetitions	1.3 (0.7 to 2.0)	1.2 (0.4 to 2.0)	1.7 (0.9 to 2.5)	−0.5 (−1.4 to 0.4)	0.29	0.40
	Delayed recall	5.2 (4.4 to 6.1)	3.5 (2.5 to 4.5)	4.8 (3.8 to 5.9)	−1.3 (−2.0 to −0.6)	0.001	NA
	Delayed intrusions	0.5 (0.2 to 0.8)	0.8 (0.1 to 1.5)	1.1 (0.4 to 1.8)	−0.3 (−1 to 0.3)	0.3	0.40
	Delayed repetitions	0.2 (0.0 to 0.3)	0.2 (−0.1 to 0.5)	0.4 (0.1 to 0.7)	−0.2 (−0.5 to 0.0)	0.07	0.19
Digit Span	Forward	5.9 (5.6 to 6.3)	5.9 (5.5 to 6.4)	6.2 (5.7 to 6.7)	−0.3 (−0.6 to 0.1)	0.12	0.25
	Reverse	3.9 (3.6 to 4.3)	3.7 (3.3 to 4.2)	3.7 (3.2 to 4.1)	0.1 (−0.4 to 0.6)	0.74	0.80

Data are mean (95% CI).

*NA* Not applicable.

PANSS-P was assessed at baseline (before CBD/placebo treatment) and after cannabis (Table 3, Fig. 1B). In the CBD arm, the mean increase was 5.0 (95% confidence interval [CI]: 3.6 to 6.5); in the placebo arm, it was 2.9 (95% CI: 1.5 to 4.3). The difference in effect between the two arms was statistically significant (estimated marginal mean difference [MD] = 2.2 [95% CI: 0.6 to 3.7]; *p* = 0.01). A large increase in PANSS-P (increase of ≥9) was observed in seven participants in the CBD arm, and in no participants in the placebo arm (*p* = 0.000005). There was no effect of visit (*p* > 0.05). Sensitivity analyses produced the same pattern of results (Tables S4 and S5).

**Table 3:** Psychopathological outcome measures.

	CBD			Placebo			CBD vs. Placebo		
	Baseline	Post-cannabis	Change	Baseline	Post-cannabis	Change	Estimated Marginal Mean difference	*P* value	Corrected *P* value
PANSS - Positive Scale	13.3 (11.3 to 15.2)	18.3 (16 to 20.6)	5.0 (3.6 to 6.5)	13.7 (11.7 to 15.6)	16.6 (14.3 to 18.9)	2.9 (1.5 to 4.3)	2.2 (0.6 to 3.7)	0.01	NA
Delusions	2.7 (2.1 to 3.4)	3.2 (2.5 to 3.9)	0.5 (0.2 to 0.8)	2.7 (2.1 to 3.4)	3.0 (2.3 to 3.7)	0.3 (0.0 to 0.6)	0.2 (−0.2 to 0.6)	0.33	0.66
Conceptual disorganization	1.3 (1.1 to 1.5)	3.5 (3 to 3.9)	2.2 (1.7 to 2.6)	1.2 (1.0 to 1.5)	2.7 (2.3 to 3.2)	1.5 (1.1 to 1.9)	0.7 (0.2 to 1.1)	0.01	0.15
Hallucinatory behaviour	2.4 (1.8 to 2.9)	2.6 (2.0 to 3.2)	0.2 (−0.3 to 0.8)	2.3 (1.7 to 2.8)	2.6 (2.0 to 3.2)	0.3 (−0.2 to 0.9)	−0.1 (−0.6 to 0.4)	0.71	0.90
Excitement	1.2 (1.0 to 1.3)	2.1 (1.6 to 2.5)	0.9 (0.5 to 1.3)	1.2 (1.1 to 1.4)	1.6 (1.2 to 2.1)	0.4 (0.0 to 0.8)	0.5 (−0.1 to 1.0)	0.09	0.43
Grandiosity	2.2 (1.6 to 2.8)	2.5 (1.9 to 3.1)	0.3 (0.1 to 0.5)	2.2 (1.7 to 2.8)	2.4 (1.8 to 3.0)	0.1 (−0.1 to 0.4)	0.2 (−0.1 to 0.4)	0.27	0.66
Suspiciousness/persecution	2.4 (1.8 to 3.0)	3.2 (2.6 to 3.7)	0.8 (0.4 to 1.2)	2.6 (2.0 to 3.2)	2.8 (2.2 to 3.4)	0.2 (−0.1 to 0.6)	0.6 (0.1 to 1.0)	0.01	0.15
Hostility	1.1 (0.9 to 1.4)	1.3 (1.0 to 1.6)	0.2 (−0.1 to 0.5)	1.4 (1.1 to 1.7)	1.4 (1.1 to 1.7)	0.0 (−0.3 to 0.3)	0.2 (−0.1 to 0.5)	0.17	0.66
PANSS - Negative Scale	13.9 (11.5 to 16.3)	17.4 (15.1 to 19.7)	3.5 (1.6 to 5.4)	14.1 (11.6 to 16.5)	16.6 (14.3 to 18.9)	2.5 (0.6 to 4.4)	1.0 (−0.9 to 2.9)	0.32	0.66
Blunted affect	2.2 (1.7 to 2.7)	3.0 (2.4 to 3.5)	0.8 (0.2 to 1.3)	2.3 (1.8 to 2.8)	2.9 (2.4 to 3.5)	0.7 (0.1 to 1.2)	0.1 (−0.4 to 0.7)	0.68	0.90
Emotional withdrawal	1.9 (1.4 to 2.3)	2.3 (1.8 to 2.8)	0.4 (0.0 to 0.8)	1.9 (1.4 to 2.4)	2.3 (1.8 to 2.8)	0.4 (0.0 to 0.9)	0.0 (−0.4 to 0.4)	0.86	0.90
Poor rapport	1.5 (1.1 to 1.9)	1.6 (1.3 to 2.0)	0.1 (−0.1 to 0.4)	1.6 (1.2 to 2.0)	1.6 (1.2 to 1.9)	0.0 (−0.3 to 0.2)	0.2 (−0.2 to 0.5)	0.31	0.66
Passive/apathetic social withdrawal	2.2 (1.6 to 2.8)	2.6 (2.0 to 3.2)	0.4 (−0.1 to 0.8)	2.2 (1.6 to 2.8)	2.4 (1.8 to 3.0)	0.2 (−0.3 to 0.7)	0.2 (−0.3 to 0.6)	0.51	0.81
Difficulty in abstract thinking	2.9 (2.4 to 3.3)	3.6 (3.1 to 4.1)	0.8 (0.3 to 1.2)	2.7 (2.2 to 3.2)	3.5 (3.0 to 4.0)	0.8 (0.3 to 1.3)	0.0 (−0.4 to 0.4)	0.93	0.93
Lack of spontaneity and flow of conversation	1.9 (1.4 to 2.3)	2.8 (2.2 to 3.3)	0.9 (0.4 to 1.4)	1.9 (1.4 to 2.3)	2.5 (2.0 to 3.0)	0.6 (0.1 to 1.1)	0.3 (−0.4 to 0.9)	0.46	0.80
Stereotyped thinking	1.4 (1.0 to 1.7)	1.5 (1.2 to 1.9)	0.2 (−0.1 to 0.4)	1.5 (1.1 to 1.9)	1.3 (1.0 to 1.7)	−0.2 (−0.4 to 0.1)	0.3 (0.0 to 0.7)	0.07	0.41
PSI - Total score	35.1 (26.4 to 43.8)	36.8 (28.5 to 45.1)	1.7 (−2.4 to 5.7)	37.5 (28.8 to 46.2)	38.5 (30.2 to 46.8)	1.1 (−3.0 to 5.1)	0.6 (−5.0 to 6.2)	0.83	0.90
Thought distortion	2.9 (1.2 to 4.7)	2.5 (1.0 to 4.0)	−0.4 (−1.4 to 0.5)	3.2 (1.5 to 4.9)	2.9 (1.4 to 4.4)	−0.3 (−1.2 to 0.6)	−0.1 (−1.4 to 1.2)	0.85	0.90
Perceptual distortion	2.3 (0.5 to 4.1)	3.2 (1.7 to 4.7)	0.9 (−0.2 to 1.9)	3.0 (1.3 to 4.8)	3.5 (2.1 to 5.0)	0.5 (−0.5 to 1.6)	0.4 (−0.9 to 1.6)	0.56	0.84
Cognitive disorganisation	3.9 (2.1 to 5.8)	5.0 (3.0 to 7.1)	1.1 (−0.4 to 2.6)	4.1 (2.3 to 6.0)	5.4 (3.3 to 7.5)	1.3 (−0.2 to 2.8)	−0.2 (−2.0 to 1.6)	0.82	0.90
Anhedonia	5.0 (3.8 to 6.2)	5.1 (3.9 to 6.3)	0.1 (−0.8 to 1.1)	5.3 (4.1 to 6.5)	5.3 (4.1 to 6.4)	0.0 (−0.9 to 0.9)	0.1 (−1.1 to 1.4)	0.83	0.90
Manic experience	4.2 (3.2 to 5.2)	3.2 (1.9 to 4.6)	−0.9 (−1.9 to 0.1)	3.9 (2.9 to 4.9)	3.9 (2.5 to 5.2)	−0.1 (−1.1 to 0.9)	−0.9 (−2.2 to 0.5)	0.23	0.66
Paranoia/suspiciousness	1.1 (−0.1 to 2.2)	1.8 (0.8 to 2.9)	0.8 (−0.2 to 1.8)	2.2 (1.0 to 3.4)	1.6 (0.5 to 2.6)	−0.6 (−1.6 to 0.4)	1.4 (0.0 to 2.7)	0.06	0.41
SSPS - Total score	10.8 (9.8 to 11.9)	10.8 (10 to 11.6)	0.0 (−1.4 to 1.3)	12.2 (11.1 to 13.2)	11.1 (10.3 to 11.9)	−1.0 (−2.3 to 0.4)	0.9 (−0.9 to 2.8)	0.33	0.66
STAI-S^a^ - Total score	43.9 (41.9 to 45.9)	42.7 (40.6 to 44.7)	−1.2 (−2.9 to 0.4)	43.9 (41.9 to 45.9)	43.5 (41.4 to 45.5)	−0.4 (−2.1 to 1.2)	−0.8 (−2.8 to 1.2)	0.43	0.80
VAS - Feel drug effect	1.7 (−2.2 to 5.7)	80.4 (72.8 to 88.1)	78.7 (69 to 88.4)	4.9 (1.0 to 8.9)	80.1 (72.5 to 87.8)	75.2 (65.5 to 84.9)	3.5 (−4.9 to 12)	0.42	0.78

Data are mean (95% CI).

^a^STAI-S was also measured pre-THC challenge. There was no difference in anxiety at this timepoint (mean difference = 0.08 [-1.33 to 1.49]; *p* = 0.91).

*PANSS* Positive and Negative Syndrome Scale, *PSI* Psychotomimetic States Inventory, *SSPS* State Social Paranoia Scale, *STAI-S* State-Trait Anxiety Inventory-State Scale, *NA* Not applicable.

Out of the seven items on the PANSS-P, two had statistically significant differences between treatment arms. For conceptual disorganisation, the mean increase in the CBD arm was 2.2 (95% CI: 1.7 to 2.6); in the placebo arm, it was 1.5 (95% CI: 1.1 to 1.9) (MD = 0.7 [95% CI: 0.2 to 1.1]; *p* = 0.01; corrected *p* = 0.15). For suspiciousness/persecution, in the CBD arm, the mean increase was 0.8 (95% CI: 0.4 to 1.2); in the placebo arm, it was 0.2 (95% CI: −0.1 to 0.6) (MD = 0.6 [95% CI: 0.1 to 1.0]; *p* = 0.01; corrected *p* = 0.15).

CBD pre-treatment was associated with greater impairment of immediate recall on the HVLT-R (*p* = 0.04), but this did not survive correction for multiple comparisons (*p* = 0.14) (Table 2). There was no effect of CBD treatment on participant rated level of intoxication, measured with a VAS for ‘Feel drug effect’ (Fig. 1D), the mean increase was 78.7 (95% CI: 69.0 to 88.4) in the CBD arm, and 75.2 (95% CI: 65.5 to 84.9) in the placebo arm (*p* = 0.42). There was no effect of CBD treatment on negative symptoms (Fig. 1C), other cognitive or psychopathological outcomes (Tables 2 and 3); or VAS (Table S6). Physiological outcome measures are presented in Table S7. There was a greater increase in systolic blood pressure in the CBD treatment group between baseline and peak (MD = 11.2 mm Hg [3.7 to 18.6]; *p* = 0.01; corrected *p* = 0.04).

#### Assessment of blinding

Participant blinding was assessed on two occasions. During the first experiment, immediately prior to THC challenge, participants correctly guessed their treatment allocation on 16/30 (53%) of occasions. At the end of the second experiment, participants correctly guessed their treatment allocation on 14/30 (47%) occasions. Researcher blinding was assessed at the second timepoint. They correctly guessed treatment allocation on 5/30 (17%) of occasions (James’ Blinding Index = 0.76 [95% CI: 0.58 to 0.94]).

### Pharmacokinetics

**Fig. 2:**
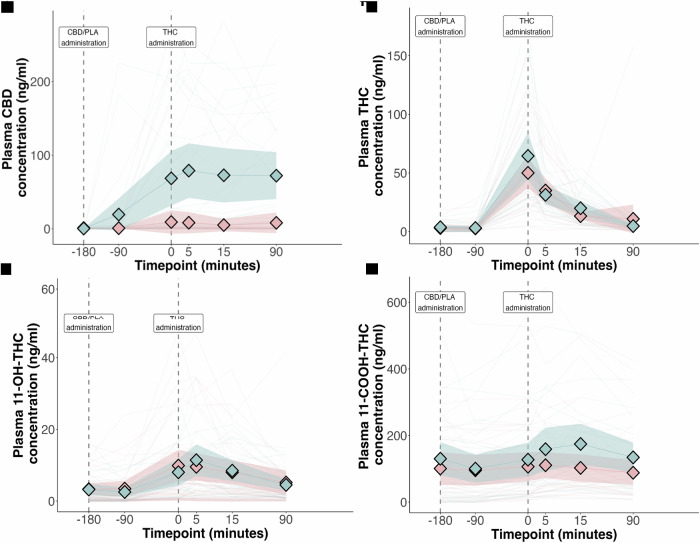
Pharmacokinetics of cannabinoids following study drug administration. Plasma concentration of (**A**) CBD; (**B**) THC; (**C**) 11-hydroxy-THC; and (**D**) 11-carboxy-THC. Data presented are arithmetic mean with 95% CI. Blue: CBD pre-treatment arm; Pink: Placebo pre-treatment arm. Shaded areas represent the 95% confidence interval.

The pharmacokinetic parameters of THC, CBD and their metabolites are presented in Table S8. We found no evidence for an effect of CBD treatment on the plasma exposure of THC, or its active metabolite 11-hydroxy-THC. CBD treatment was associated with increased plasma exposure of 11-carboxy-THC. The plasma concentration-time profile of THC and CBD are presented in Fig. 2A-D. There was a statistically significant correlation between CBD AUC and change in PANSS-P (*r* = 0.43 [0.19 to 0.61], *p* = 0.018) and HVLT delayed recall (*r* = −0.27 [−0.49 to −0.02], *p* = 0.038). There was no significant correlation between the plasma concentration of THC or 11-hydroxy-THC and delayed verbal recall or change in PANSS-P (Table S9 and Fig. S4).

### Discussion

To our knowledge, this is the first study to investigate if CBD can moderate the acute effects of cannabis in people with either schizophrenia or a cannabis use disorder. Our main finding was that CBD increased severity of verbal memory impairment and positive psychotic symptoms following cannabis administration. Our findings were not attributable to a pharmacokinetic interaction between CBD and THC.

Some previous studies in healthy volunteers have found that CBD increases the acute adverse effects of THC when they are co-administered orally [34–36]. However, this is because CBD inhibits the hepatic metabolism of THC through the inhibition of CYP enzymes [34]. In the present study, pharmacokinetic analyses indicated that CBD pre-treatment did not affect the plasma exposure of THC or its pharmacologically active metabolite, 11-hydroxy-THC. Furthermore, in a post-hoc analysis, there was a significant correlation between CBD plasma concentration and positive psychotic symptoms and memory impairment, correlations that were not evident with either THC or 11-hydroxy-THC. These findings suggest that the exacerbation of THC’s effects by CBD was unlikely to be the result of a pharmacokinetic mechanism, and may have been due to a pharmacodynamic effect.

Our results contrast with data from two studies in healthy volunteers which reported that pre-treatment with CBD reduced the adverse effects of THC [32, 33]. However, both schizophrenia and cannabis use disorder are associated with significant alterations in the brain endocannabinoid system [11, 47, 48], as well as mitochondrial function [49, 50], which could modify the effects of CBD and THC. In the present study, we chose to examine a subgroup of patients with schizophrenia who also had a CUD, because we were interested in assessing the potential of CBD as a treatment for patients with schizophrenia whose symptoms are exacerbated by cannabis use. As a result, it is unclear whether the differences between the present findings and those previously reported in healthy volunteers are related to an effect of schizophrenia, of CUD, or both. Most patients with schizophrenia do not have CUD [4], and future studies could investigate whether the effects we observed are also evident in patients who don’t use cannabis, or only do so occasionally. Similarly, it would be useful to repeat our investigation in people who have a CUD but do not have schizophrenia.

CBD is a candidate novel treatment for schizophrenia: a number of trials suggest that it can reduce psychotic symptoms in patients with the disorder, and in people at clinical high risk for the disorder [24, 25, 27]. It is important to emphasise that the results of the present study relate to effects of CBD on psychotic symptoms experimentally induced by THC, rather than symptoms of schizophrenia itself. Moreover, our study involved participants with both schizophrenia and a CUD, as opposed to schizophrenia alone. Clinical trials investigating CBD as a treatment for schizophrenia should compare its efficacy and safety in individuals with and without comorbid CUD.

Strengths of our study include the use of a high oral dose of CBD, equivalent to the oral dose used in clinical trials of schizophrenia, and the administration of THC via inhalation, the route employed by most recreational cannabis users. The incorporation of a THC dose-ranging approach reduced the proportion of participants who did not show a symptomatic response to cannabis, increasing our ability to detect a modulatory effect of CBD. One limitation is that participants were administered a fixed dose of THC, rather than titrating their intake according to their desired level of intoxication. It is therefore unclear whether the effects associated with CBD in this study would occur in a real-world setting. Other limitations are that we only investigated a single dose of CBD and that the sample was predominantly male. Finally, the crossover design, pre-registered statistical analysis plan, and collection of pharmacokinetic data strengthen the internal validity of the study.

Our findings do not support the use of CBD as a means of ameliorating the acute adverse effects of cannabis in people with schizophrenia. However, because we studied patients with comorbid CUD, we cannot exclude the possibility that CBD might reduce the effects of cannabis in patients with schizophrenia who do not have a comorbid CUD.

## Supplementary information

Supplementary materials
