# Supplementary material for: Does cannabidiol reduce the adverse effects of cannabis in schizophrenia? A randomised, double-blind, cross-over trial
Source: Neuropsychopharmacology. 2025 Jul 24;50(12):1759–67. doi: 10.1038/s41386-025-02175-3 (PMC12518716; doi:10.1038/s41386-025-02175-3)

## Supplementary Materials

Figure S1. Study Design

Figure S2. Trial procedures and study timeline

Figure S3. Consort flow diagram

Table S1. Demographic and clinical characteristics of the intention-to-treat, per-protocol and higher THC dose populations

Table S2. Cognitive outcome measures: intention-to-treat sensitivity analysis

Table S3. Cognitive outcome measures: standard dose sensitivity analyses

Table S4. PANSS: intention-to-treat sensitivity analysis

Table S5. PANSS: standard dose sensitivity analyses

Table S6. Visual analogue scales

Table S7. Physiological outcome measures

Table S8. Pharmacokinetic parameters of THC, CBD and metabolites

Table S9. Correlation between plasma exposure of CBD, THC and 11-OH-THC and change in PANSS-P and Hopkins Verbal Learning Task – Delayed Recall.

Figure S4. Correlation between plasma exposure of CBD, THC and 11-OH-THC and change in PANSS-P and Hopkins Verbal Learning Task – Delayed Recall.

Figure S1. Study design

Figure S2. Trial procedures and study timeline. PANSS: Positive and Negative Syndrome Scale; STAI: State-Trait Anxiety Inventory; PSI: Psychotomimetic States Inventory; SSPS: State Social Paranoia Scale; HVLT: Hopkins Verbal Learning Task; VAS: Visual Analogue Scales.

Figure S3. CONSORT flow diagram

| **Table S1. Demographic and clinical characteristics of the intention-to-treat, per-protocol and higher THC dose populations** | | | | |
| --- | --- | --- | --- | --- |
|  |  | Intention-to-treat (n=34) | Per-protocol (n=30) | Higher THC dose (n=6) |
|  | Age (years) | 40.2 (11.2) | 39.7 (11.1) | 40.8 (8.4) |
| Sex | Male | 32 (94.1%) | 28 (93.3%) | 5 (83.3%) |
|  | Female | 2 (5.9%) | 2 (6.7%) | 1 (16.7%) |
| Ethnicity | Black | 18 (52.9%) | 17 (56.7%) | 3 (50.0%) |
|  | Mixed/Other | 7 (20.5%) | 7 (23.3%) | 1 (16.7%) |
|  | White | 9 (26.4%) | 6 (20.0%) | 2 (33.3%) |
| Employed | Yes | 3 (8.8%) | 3 (10.0%) | 0 (0%) |
|  | No | 31 (91.2%) | 27 (90.0%) | 6 (100%) |
| Primary diagnosis (ICD-10 criteria) | Schizophrenia | 31 (91.2%) | 27 (90.0%) | 4 (66.7%) |
|  | Schizoaffective disorder | 3 (8.8%) | 3 (10.0%) | 2 (33.3%) |
| Illness duration and severity | Years of illness | 17.2 (11.0) | 17.1 (11.5) | 18.0 (10.3) |
|  | Relapses in past 10 years | 2.8 (3.0) | 2.6 (2.5) | 4.2 (2.2) |
| Treatment and management | Olanzapine equivalents | 13.9 (7.8) | 14.4 (7.7) | 8.9 (3.0) |
|  | Long-acting injectable (current) | 27 (79.4%) | 25 (83.3%) | 6 (100%) |
|  | Clozapine (current) | 4 (11.7%) | 4 (13.3%) | 0 (0.0%) |
|  | Community Treatment Order (ever) | 8 (23.5%) | 7 (23.3%) | 3 (50.0%) |
| Baseline Symptom severity | Positive subscale | 14.0 (5.7) | 13.9 (5.5) | 12.7 (5.1) |
|  | Negative subscale | 13.5 (6.6) | 13.3 (6.0) | 15.2 (9.0) |
|  | AUDIT score | 4.2 (5.1) | 4.3 (4.2) | 4.5 (4.0) |
|  | CUDIT score | 15.6 (4.9) | 15.9 (4.9) | 14.7 (6.7) |
| Cannabis Use Disorder (DSM-5) | Mild | 1 (2.9%) | 1 (3.3%) | 0 (0.0%) |
|  | Moderate | 2 (5.9%) | 2 (6.7%) | 0 (0.0%) |
|  | Severe | 31 (91.2%) | 27 (90.0%) | 6 (100%) |
| Cannabis use | Days per week | 5.4 (1.9) | 5.4 (1.9) | 5.8 (1.8) |
|  | Joints per day | 5.4 (4.3) | 5.3 (4.2) | 7.0 (6.6) |
|  | Grams per day | 1.0 (0.7) | 1.1 (0.7) | 1.6 (1.1) |
|  | High potency | 28 (82.3%) | 24 (80.0%) | 6 (100%) |
|  | Low potency | 6 (17.6%) | 6 (20.0%) | 0 (0%) |
|  | Tobacco in joints | 34 (100%) | 30 (100%) | 6 (100%) |
| Tobacco use* | Yes | 29 (85.3%) | 25 (83.3%) | 6 (100%) |
|  | No | 5 (14.7%) | 5 (17.6%) | 0 (0%) |
| Data are n (%) or mean (SD)  DSM-5: Diagnostic and Statistical Manual of Mental Disorders, Fifth Edition; AUDIT: Alcohol use disorders identification test; CUDIT: Cannabis use disorder identification test.  *Independent of cannabis use | | | | |

| **Table S2. Cognitive outcome measures: intention-to-treat sensitivity analysis (n=34)** | | | | | | | |
| --- | --- | --- | --- | --- | --- | --- | --- |
|  |  |  | CBD | Placebo | Comparison |  |  |
|  |  | Screening visit | Post-cannabis | Post-cannabis | Mean difference | P value | Corrected P value |
| HVLT - Revised | Immediate recall | 19.0 (17.0-21.0) | 15.0 (13.0-17.0) | 16.6 (14.6-18.6) | -1.6 (-3.2-0.0) | 0.065 | 0.28 |
|  | Immediate intrusions | 0.9 (0.6-1.2) | 2.3 (1.2-3.4) | 2.1 (1.0-3.2) | 0.2 (-0.7-1.1) | 0.71 | 0.71 |
|  | Immediate repetitions | 1.4 (0.8-2.0) | 1.2 (0.4-1.9) | 1.7 (1.0-2.4) | -0.5 (-1.3-0.3) | 0.26 | 0.42 |
|  | Delayed recall | 5.2 (4.4-6.1) | 3.3 (2.3-4.2) | 4.6 (3.6-5.5) | -1.3 (-2.0--0.6) | 0.001 | NA |
|  | Delayed intrusions | 0.5 (0.2-0.8) | 0.7 (0.1-1.4) | 1.1 (0.4-1.7) | -0.3 (-0.9-0.3) | 0.30 | 0.42 |
|  | Delayed repetitions | 0.2 (0.0-0.3) | 0.1 (-0.2-0.4) | 0.4 (0.1-0.6) | -0.2 (-0.5-0.0) | 0.081 | 0.28 |
|  |  |  |  |  |  |  |  |
| Digit Span | Forward | 5.9 (5.5-6.3) | 6.0 (5.6-6.5) | 6.3 (5.9-6.8) | -0.3 (-0.6-0.1) | 0.12 | 0.29 |
|  | Reverse | 4.0 (3.6-4.3) | 3.8 (3.4-4.3) | 3.7 (3.3-4.2) | 0.1 (-0.4-0.6) | 0.69 | 0.71 |
| Data are mean (95% CI) | | | | | | | |

| **Table S3. Cognitive outcome measures: standard dose sensitivity analyses (20 mg sessions only; per-protocol; n=30)** | | | | | | | |
| --- | --- | --- | --- | --- | --- | --- | --- |
|  |  |  | CBD | Placebo | Comparison |  |  |
|  |  | Screening visit | Post-cannabis | Post-cannabis | Mean difference | P value | Corrected P value |
| HVLT - Revised | Immediate recall | 19.3 (17.2-21.4) | 15.2 (13.0-17.4) | 17.0 (14.7-19.2) | -1.8 (-3.3--0.2) | 0.04 | 0.25 |
|  | Immediate intrusions | 0.9 (0.6-1.2) | 2.1 (0.9-3.2) | 2.1 (0.9-3.2) | 0.0 (-1.0-1.0) | 1.00 | 1.00 |
|  | Immediate repetitions | 1.3 (0.7-2.0) | 1.6 (0.7-2.5) | 1.6 (0.6-2.5) | 0.0 (-1.0-1.0) | 0.95 | 1.00 |
|  | Delayed recall | 5.2 (4.4-6.1) | 3.5 (2.5-4.5) | 4.7 (3.7-5.7) | -1.3 (-2.0--0.6) | 0.002 | NA |
|  | Delayed intrusions | 0.5 (0.2-0.8) | 1.1 (0.3-1.9) | 1.0 (0.2-1.8) | 0.2 (-0.6-1.0) | 0.69 | 1.00 |
|  | Delayed repetitions | 0.2 (0.0-0.3) | 0.3 (0.0-0.5) | 0.3 (0.0-0.5) | 0.0 (-0.3-0.3) | 1.00 | 1.00 |
|  |  |  |  |  |  |  |  |
| Digit Span | Forward | 5.9 (5.6-6.3) | 5.9 (5.4-6.4) | 6.1 (5.6-6.6) | -0.2 (-0.6-0.2) | 0.28 | 0.65 |
|  | Reverse | 3.9 (3.6-4.3) | 3.6 (3.0-4.3) | 4.1 (3.5-4.7) | -0.5 (-1.3-0.3) | 0.26 | 0.65 |
| Data are mean (95% CI) | | | | | | | |

| **Table S4. Positive psychotic symptoms: intention-to-treat sensitivity analysis (n=34)** | | | | | | | | | |
| --- | --- | --- | --- | --- | --- | --- | --- | --- | --- |
|  | CBD |  |  | Placebo |  |  | Comparison |  |  |
| PANSS - Positive Scale | Baseline | Post-cannabis | Change | Baseline | Post-cannabis | Change | Estimated Marginal Mean difference | P value | Corrected P value |
| Total Score | 13.3 (11.4-15.1) | 18.8 (16.5-21.1) | 5.6 (4.1-7.2) | 13.7 (11.8-15.5) | 17.0 (14.8-19.2) | 3.2 (1.7-4.7) | 2.4 (0.8-4.0) | 0.0059 | NA |
| Delusions | 2.8 (2.2-3.4) | 3.3 (2.7-4.0) | 0.6 (0.3-1.0) | 2.8 (2.2-3.4) | 3.1 (2.5-3.8) | 0.2 (-0.1-0.6) | 0.4 (-0.1-0.8) | 0.14 | 0.23 |
| Conceptual disorganization | 1.4 (1.1-1.6) | 3.5 (3.1-4.0) | 2.2 (1.8-2.6) | 1.3 (1.1-1.5) | 2.8 (2.3-3.2) | 1.5 (1.1-1.9) | 0.7 (0.3-1.2) | 0.0050 | 0.03 |
| Hallucinatory behaviour | 2.3 (1.8-2.8) | 2.7 (2.1-3.3) | 0.3 (-0.2-0.9) | 2.2 (1.7-2.7) | 2.6 (2.0-3.1) | 0.4 (-0.2-0.9) | -0.1 (-0.6-0.4) | 0.82 | 0.82 |
| Excitement | 1.2 (1.0-1.3) | 2.2 (1.7-2.6) | 1.0 (0.6-1.4) | 1.2 (1.0-1.4) | 1.7 (1.3-2.2) | 0.5 (0.1-0.9) | 0.5 (0.1-1.0) | 0.078 | 0.18 |
| Grandiosity | 2.2 (1.6-2.7) | 2.4 (1.8-3.0) | 0.3 (0.0-0.5) | 2.2 (1.7-2.7) | 2.3 (1.7-2.8) | 0.1 (-0.2-0.3) | 0.2 (-0.1-0.5) | 0.16 | 0.23 |
| Suspiciousness/persecution | 2.4 (1.8-2.9) | 3.3 (2.8-3.9) | 1.0 (0.6-1.4) | 2.6 (2.0-3.1) | 3.0 (2.4-3.6) | 0.4 (0.0-0.8) | 0.6 (0.2-1.0) | 0.010 | 0.04 |
| Hostility | 1.1 (0.9-1.4) | 1.4 (1.1-1.7) | 0.2 (-0.1-0.5) | 1.4 (1.2-1.7) | 1.5 (1.2-1.8) | 0.1 (-0.2-0.3) | 0.2 (-0.1-0.4) | 0.25 | 0.29 |
| Data are mean (95% CI) | | | | | | | | | |

| **Table S5. Positive psychotic symptoms: standard dose sensitivity analyses (20 mg sessions only; per-protocol; n=30)** | | | | | | | | | |
| --- | --- | --- | --- | --- | --- | --- | --- | --- | --- |
|  | CBD |  |  | Placebo |  |  | Comparison |  |  |
| PANSS - Positive Scale | Baseline | Post-cannabis | Change | Baseline | Post-cannabis | Change | Estimated Marginal Mean difference | P value | Corrected P value |
| Total Score | 13.2 (11.3-15.2) | 17.8 (15.4-20.1) | 4.5 (3.0-6.0) | 13.7 (11.7-15.6) | 16.1 (13.8-18.5) | 2.5 (1.0-4.0) | 2.1 (0.6-3.6) | 0.012 | NA |
| Delusions | 2.8 (2.1-3.5) | 3.2 (2.6-3.9) | 0.4 (0.2-0.7) | 2.8 (2.1-3.5) | 2.9 (2.2-3.5) | 0.1 (-0.2-0.3) | 0.4 (0.1-0.7) | 0.025 | 0.09 |
| Conceptual disorganization | 1.3 (1.1-1.5) | 3.2 (2.7-3.7) | 2.0 (1.5-2.4) | 1.2 (1.0-1.4) | 2.7 (2.2-3.2) | 1.5 (1.0-2.0) | 0.5 (0.0-0.9) | 0.064 | 0.13 |
| Hallucinatory behaviour | 2.3 (1.7-2.8) | 2.4 (1.8-2.9) | 0.1 (-0.5-0.7) | 2.2 (1.7-2.8) | 2.5 (1.9-3.1) | 0.3 (-0.3-0.8) | -0.2 (-0.6-0.3) | 0.46 | 0.46 |
| Excitement | 1.2 (1.0-1.4) | 2.0 (1.6-2.4) | 0.8 (0.4-1.2) | 1.2 (1.0-1.4) | 1.5 (1.1-2.0) | 0.3 (-0.1-0.7) | 0.5 (0.0-1.0) | 0.075 | 0.13 |
| Grandiosity | 2.2 (1.6-2.8) | 2.5 (1.9-3.1) | 0.3 (0.0-0.5) | 2.2 (1.7-2.8) | 2.3 (1.7-2.9) | 0.1 (-0.1-0.3) | 0.2 (-0.1-0.4) | 0.22 | 0.30 |
| Suspiciousness/persecution | 2.4 (1.8-3.0) | 3.1 (2.5-3.8) | 0.8 (0.4-1.1) | 2.6 (2.0-3.2) | 2.8 (2.2-3.4) | 0.2 (-0.2-0.5) | 0.6 (0.2-1.0) | 0.0042 | 0.03 |
| Hostility | 1.1 (0.9-1.4) | 1.3 (1.0-1.6) | 0.2 (-0.1-0.5) | 1.3 (1.1-1.5) | 1.4 (1.1-1.7) | 0.1 (-0.2-0.3) | 0.1 (-0.1-0.4) | 0.30 | 0.35 |
| Data are mean (95% CI) | | | | | | | | | |

| **Table S6. Visual analogue scales** | | | | | | | | |
| --- | --- | --- | --- | --- | --- | --- | --- | --- |
|  | CBD |  |  | Placebo |  |  | Comparison |  |
| Item | Baseline | Post-cannabis | Change | Baseline | Post-cannabis | Change | Mean difference | P value |
| Feel drug effect | 1.7 (-2.2-5.7) | 80.4 (72.8-88.1) | 78.7 (69.0-88.4) | 4.9 (1.0-8.9) | 80.1 (72.5-87.8) | 75.2 (65.5-84.9) | 3.5 (-4.9-12.0) | 0.42 |
| Like drug effect | 10.5 (1.2-19.8) | 72.6 (62.7-82.4) | 62.0 (48.1-75.9) | 16.8 (7.5-26.1) | 73.0 (63.1-82.8) | 56.2 (42.3-70.1) | 5.8 (-7.9-19.5) | 0.41 |
| Want more drug | 37.7 (24.2-51.1) | 49.3 (36.6-62.1) | 11.7 (-2.4-25.7) | 34.7 (21.3-48.1) | 55.2 (42.4-67.9) | 20.5 (6.5-34.5) | -8.9 (-27.8-10.1) | 0.37 |
| Thinking clearly | 78.6 (68.0-89.3) | 64.1 (54.4-73.9) | -14.5 (-26.5--2.4) | 70.3 (59.6-80.9) | 66.1 (56.3-75.9) | -4.2 (-16.2-7.9) | -10.3 (-23.4-2.8) | 0.13 |
| Tired | 22.4 (13.5-31.2) | 30.5 (21.0-40.0) | 8.1 (-4.4-20.6) | 25.2 (16.3-34.0) | 31.1 (21.6-40.6) | 5.9 (-6.6-18.4) | 2.2 (-14.3-18.7) | 0.80 |
| Want to talk | 38.7 (29.4-47.9) | 49.3 (38.5-60.2) | 10.6 (-2.0-23.2) | 42.9 (33.6-52.2) | 51.9 (41.0-62.7) | 9.0 (-3.5-21.6) | 1.6 (-10.7-13.8) | 0.81 |
| Anxious | 11.1 (3.9-18.3) | 19.3 (11.0-27.6) | 8.2 (-2.3-18.6) | 15.8 (8.6-23.0) | 18.3 (10.1-26.6) | 2.6 (-7.9-13.0) | 5.6 (-7.6-18.8) | 0.41 |
| Suspicious | 8.3 (2.6-14.1) | 11.1 (6.6-15.7) | 2.8 (-3.9-9.6) | 8.2 (2.5-14.0) | 8.4 (3.9-13.0) | 0.2 (-6.6-6.9) | 2.7 (-6.6-11.9) | 0.58 |
| Happy | 59.0 (50.9-67.2) | 75.5 (68.2-82.8) | 16.5 (7.8-25.2) | 64.0 (55.8-72.1) | 72.1 (64.8-79.4) | 8.1 (-0.6-16.8) | 8.4 (-2.5-19.2) | 0.14 |
| Irritable | 7.5 (2.3-12.8) | 7.1 (1.2-13.0) | -0.5 (-6.9-5.9) | 12.1 (6.8-17.4) | 14.9 (8.9-20.8) | 2.8 (-3.6-9.2) | -3.2 (-11.8-5.4) | 0.47 |
| Relaxed | 61.6 (52.2-70.9) | 73.3 (66.2-80.5) | 11.8 (1.5-22.1) | 66.5 (57.1-75.9) | 73.3 (66.1-80.4) | 6.8 (-3.5-17.1) | 5.0 (-9.0-18.9) | 0.49 |
| Hearing voices | 7.4 (2.7-12.2) | 11.4 (3.9-19.0) | 4.0 (-3.9-11.9) | 5.5 (0.8-10.3) | 13.0 (5.4-20.5) | 7.4 (-0.5-15.3) | -3.4 (-11.2-4.3) | 0.39 |
| Dry mouth | 15.9 (6.9-24.9) | 33.4 (22.7-44.1) | 17.5 (5.3-29.7) | 17.8 (8.8-26.8) | 23.8 (13.1-34.5) | 6.0 (-6.2-18.2) | 11.6 (-2.7-25.8) | 0.12 |
| Vulnerable | 15.4 (6.5-24.2) | 19.4 (10.3-28.5) | 4.0 (-6.4-14.3) | 15.7 (6.8-24.5) | 17.1 (8.0-26.2) | 1.4 (-8.9-11.8) | 2.5 (-11.0-16.1) | 0.72 |
| Threatened | 5.8 (1.3-10.2) | 22.4 (13.8-31.0) | 16.6 (7.4-25.9) | 7.7 (3.3-12.2) | 23.6 (15-32.3) | 15.9 (6.6-25.2) | 0.8 (-9.2-10.7) | 0.88 |
| Excited | 47.7 (37.1-58.3) | 4.9 (1.4-8.5) | -42.7 (-54.1--31.4) | 49.2 (38.6-59.8) | 6.5 (3.0-10.0) | -42.7 (-54.1--31.3) | -0.1 (-12.3-12.2) | 0.99 |
| Data are mean (95% CI) | | | | | | | | |

| **Table S7. Physiological outcome measures** | | | | | | | | | |
| --- | --- | --- | --- | --- | --- | --- | --- | --- | --- |
|  | CBD |  |  | Placebo |  |  |  |  |  |
|  | Baseline | Peak post-cannabis | Change | Baseline | Peak post-cannabis | Change | Estimated Marginal Mean difference | P value | Corrected P value |
| Pulse (bpm) | 76.9 (72.2-81.6) | 103.4 (95.2-111.7) | 26.6 (19.2-34.0) | 76.3 (71.6-81.0) | 103.3 (95.1-111.5) | 27.0 (19.6-34.3) | -0.4 (-6.5-5.8) | 0.91 | 0.91 |
| Systolic BP (mm Hg) | 121.2 (117.3-125.1) | 146.2 (139.9-152.4) | 25.1 (19.5-30.8) | 122.3 (118.5-126.1) | 136.5 (130.3-142.6) | 13.9 (8.4-19.5) | 11.2 (3.7-18.6) | 0.01 | 0.04 |
| Diastolic BP (mm Hg) | 76.3 (72.9-79.7) | 95.2 (89.3-101.1) | 18.9 (12.8-25) | 75.6 (72.3-79.0) | 89.6 (83.8-95.4) | 13.9 (8-19.9) | 5.0 (-1.7-11.7) | 0.16 | 0.28 |
| Temperature (^o^C) | 36.3 (36.1-36.5) | 36.8 (36.7-37.0) | 0.5 (0.4-0.7) | 36.4 (36.2-36.6) | 36.8 (36.6-36.9) | 0.4 (0.2-0.5) | 0.1 (-0.1-0.3) | 0.21 | 0.28 |
| Data are mean (95% CI) | | | | | | | | | |

| **Table S8. Pharmacokinetic parameters of THC, CBD and metabolites** | | | | |
| --- | --- | --- | --- | --- |
| Pharmacokinetic parameter (unit) | CBD/THC arm | Placebo/THC arm | p value |  |
| CBD |  |  |  |  |
| AUC_Baseline-90 min_ (ng·h/mL) | 2514.2 (1065.0-5935.5) | 161.0 (91.7-282.7) | 0.0006 |  |
| C_max_ (ng/mL) | 25.7 (11.1-59.5) | 1.7 (1.0-2.9) | 0.0003 |  |
| 6-hydroxy-CBD |  |  |  |  |
| AUC_Baseline-90 min_ (ng·h/mL) | 0.1 (0.0-4.1) | 0.0 (0.0-0.0) | 0.008 |  |
| C_max_ (ng/mL) | 0.0 (0.0-0.2) | 0.0 (0.0-0.0) | 0.002 |  |
| 7-hydroxy-CBD |  |  |  |  |
| AUC_Baseline-90 min_ (ng·h/mL) | 200.8 (17.7-2273.9) | 10.4 (1.4-78.1) | 0.004 |  |
| C_max_ (ng/mL) | 3.1 (0.4-22.8) | 0.2 (0.0-0.9) | 0.002 |  |
| 7-carboxy-CBD |  |  |  |  |
| AUC_Baseline-90 min_ (ng·h/mL) | 3083.4 (1477.8-6433.4) | 421.3 (230.6-769.9) | 0.0039 |  |
| C_max_ (ng/mL) | 29.2 (13.6-62.4) | 3.5 (1.9-6.4) | 0.0008 |  |
| THC |  |  |  |  |
| AUC_0-90 min_ (ng·h/mL) | 984.2 (769.7-1258.5) | 973.1 (756.1-1252.5) | 0.81 |  |
| C_max_ (ng/mL) | 45.7 (33.1-63) | 37.2 (28.0-49.4) | 0.12 |  |
| 11-hydroxy-THC |  |  |  |  |
| AUC_0-90 min_ (ng·h/mL) | 439.8 (304.9-634.3) | 398.0 (281.8-562.2) | 0.85 |  |
| C_max_ (ng/mL) | 9.4 (6.5-13.6) | 7.9 (5.6-11.2) | 0.55 |  |
| 11-carboxy-THC |  |  |  |  |
| AUC_0-90 min_ (ng·h/mL) | 7130.8 (4849.4-10485.5) | 5171.8 (3596.7-7436.8) | 0.015 |  |
| C_max_ (ng/mL) | 140.2 (99.6-197.2) | 90.5 (63.9-128.3) | 0.001 |  |
| All parameters are reported as geometric mean (geometric CV%). | | | | |

| **Table S9. Correlation between area under the curve (AUC) of CBD, THC and 11-OH-THC and change in PANSS-P and Hopkins Verbal Learning Task – Delayed Recall** | | | | |
| --- | --- | --- | --- | --- |
| Metabolite | PANSS-P | P value | Delayed verbal recall | P value |
| CBD | 0.43 (0.19 to 0.61) | 0.018 | -0.27 (-0.49 to -0.02) | 0.038 |
| THC | 0.01 (-0.27 to 0.30) | 0.92 | 0.10 (-0.19 to 0.37) | 0.50 |
| 11-OH-THC | -0.01 (-0.27 to 0.24) | 0.93 | 0.10 ( -0.16 to 0.34) | 0.45 |

Figure S4. Correlation between plasma exposure of CBD, THC and 11-OH-THC and change in PANSS-P and Hopkins Verbal Learning Task – Delayed Recall. Shaded area = Standard Error.


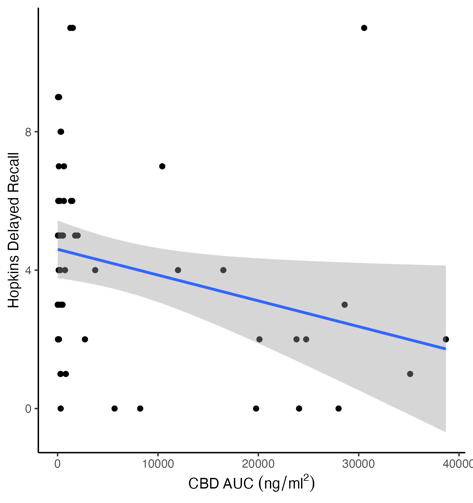

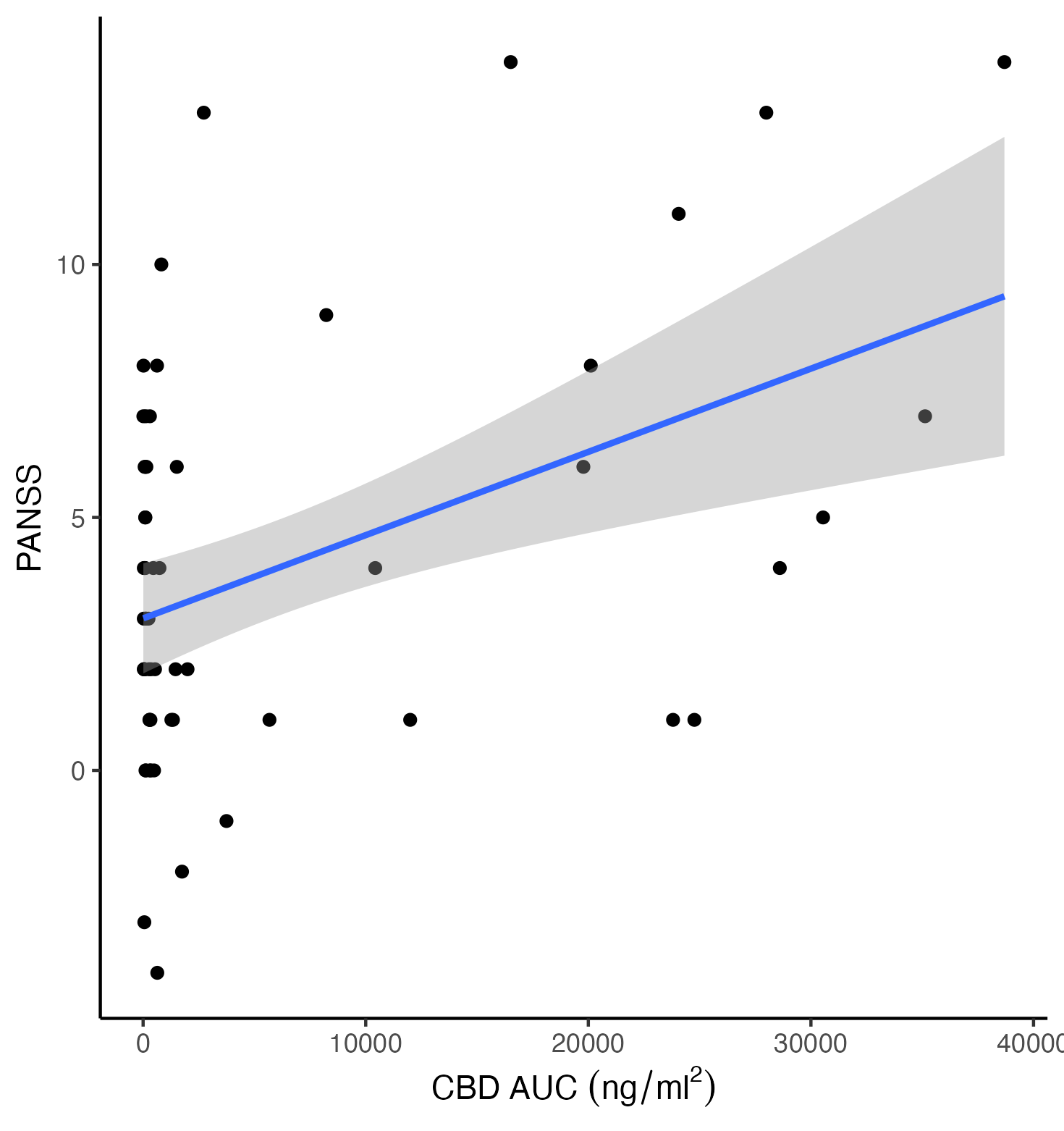

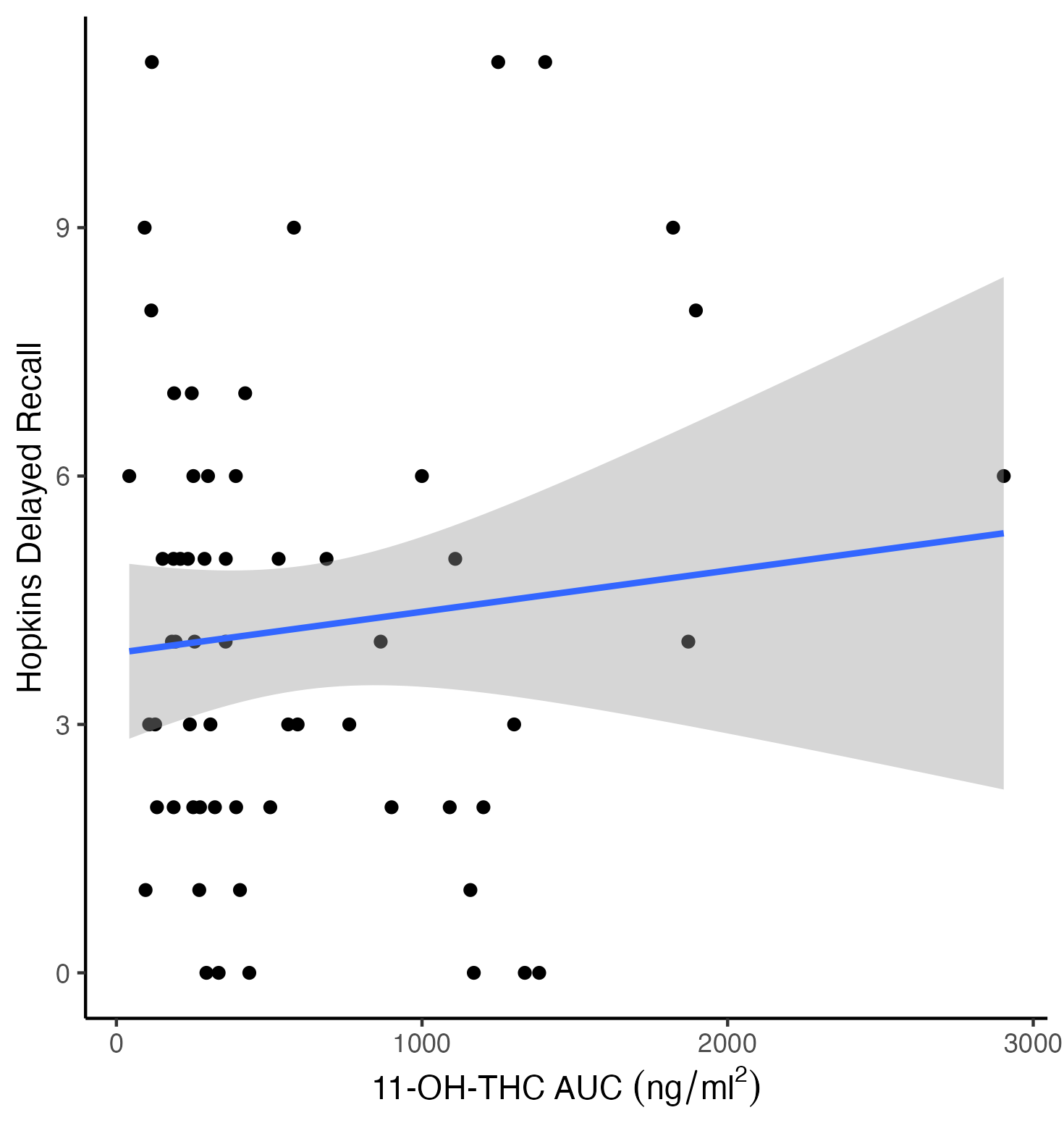

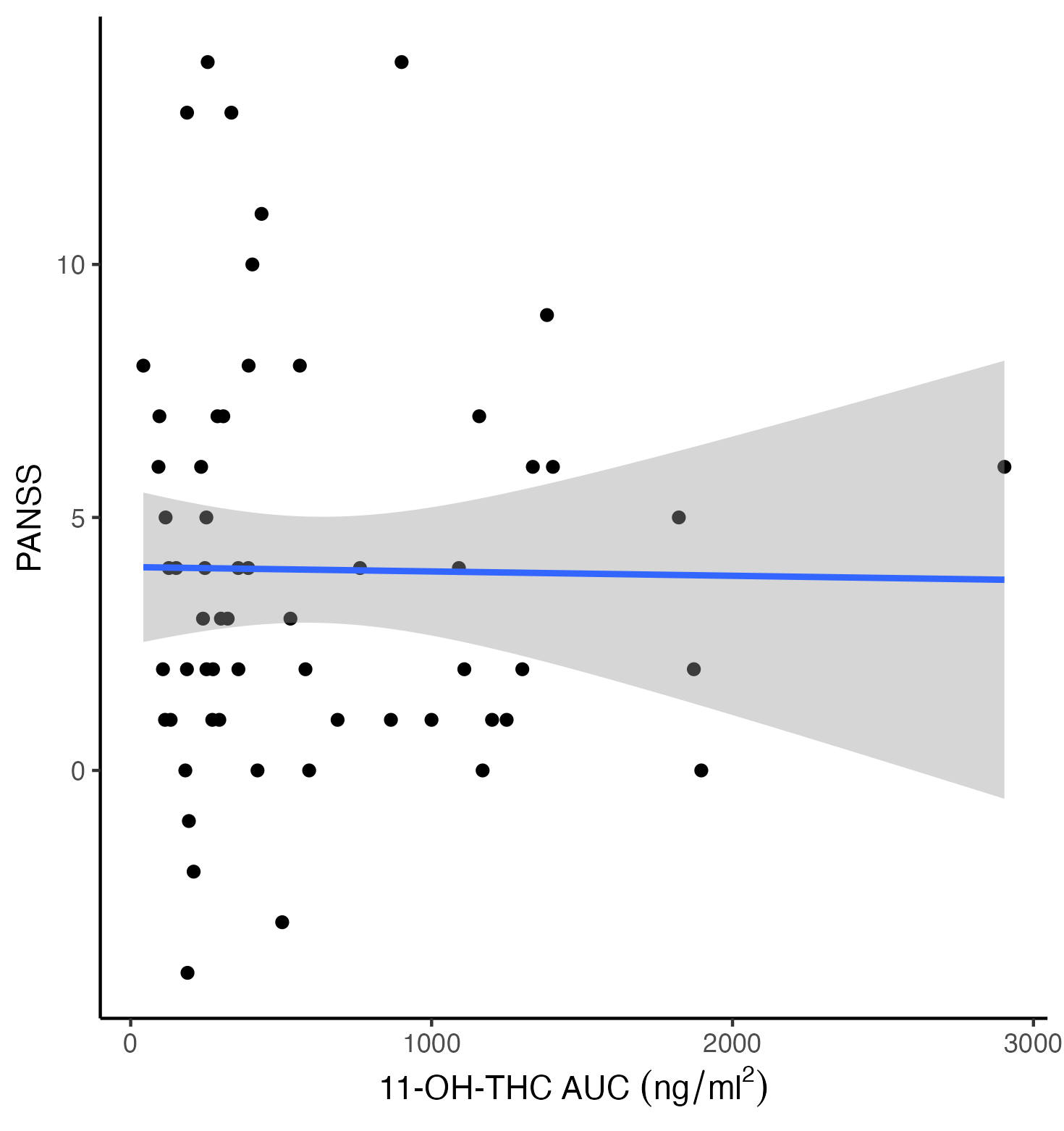

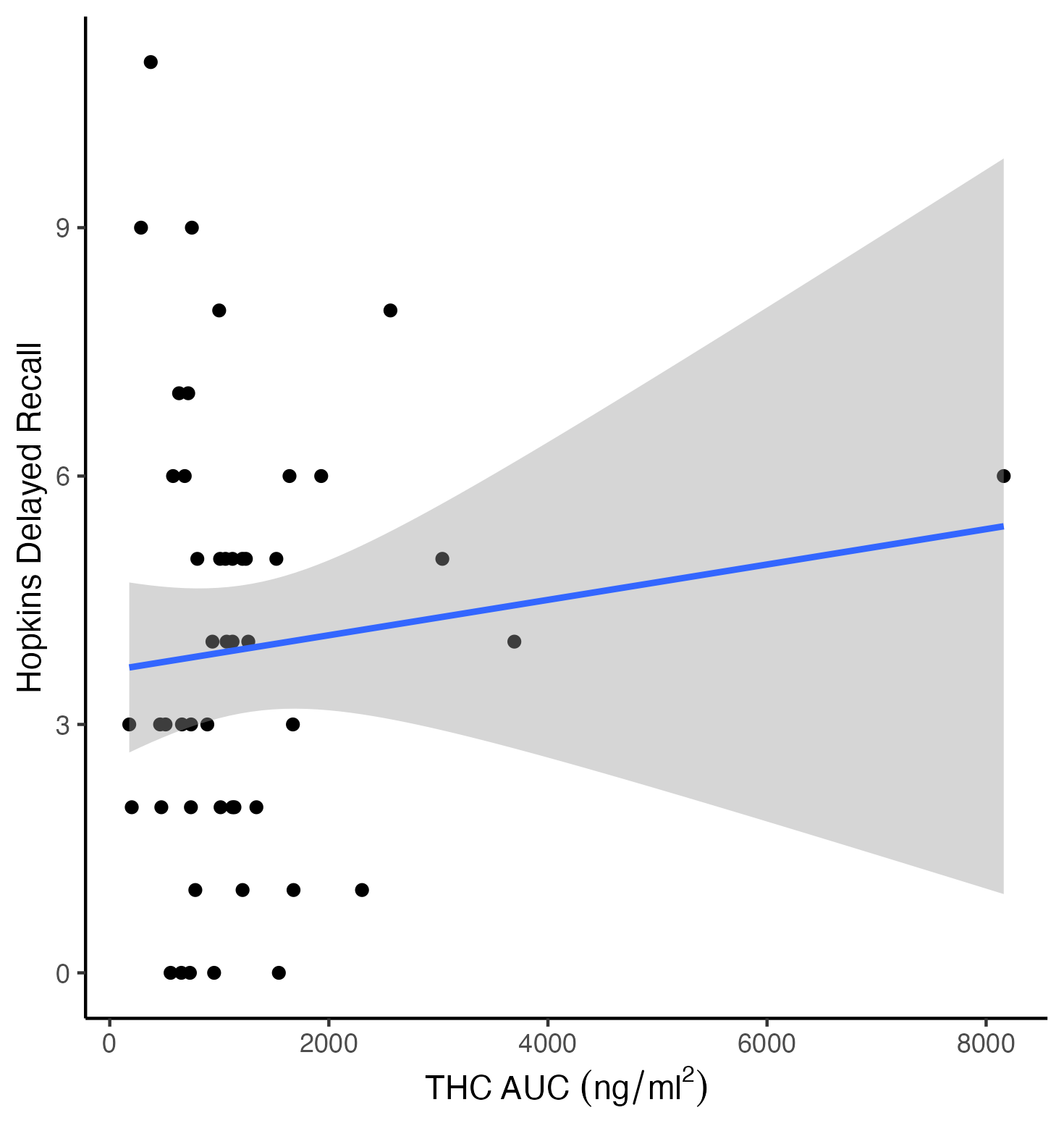

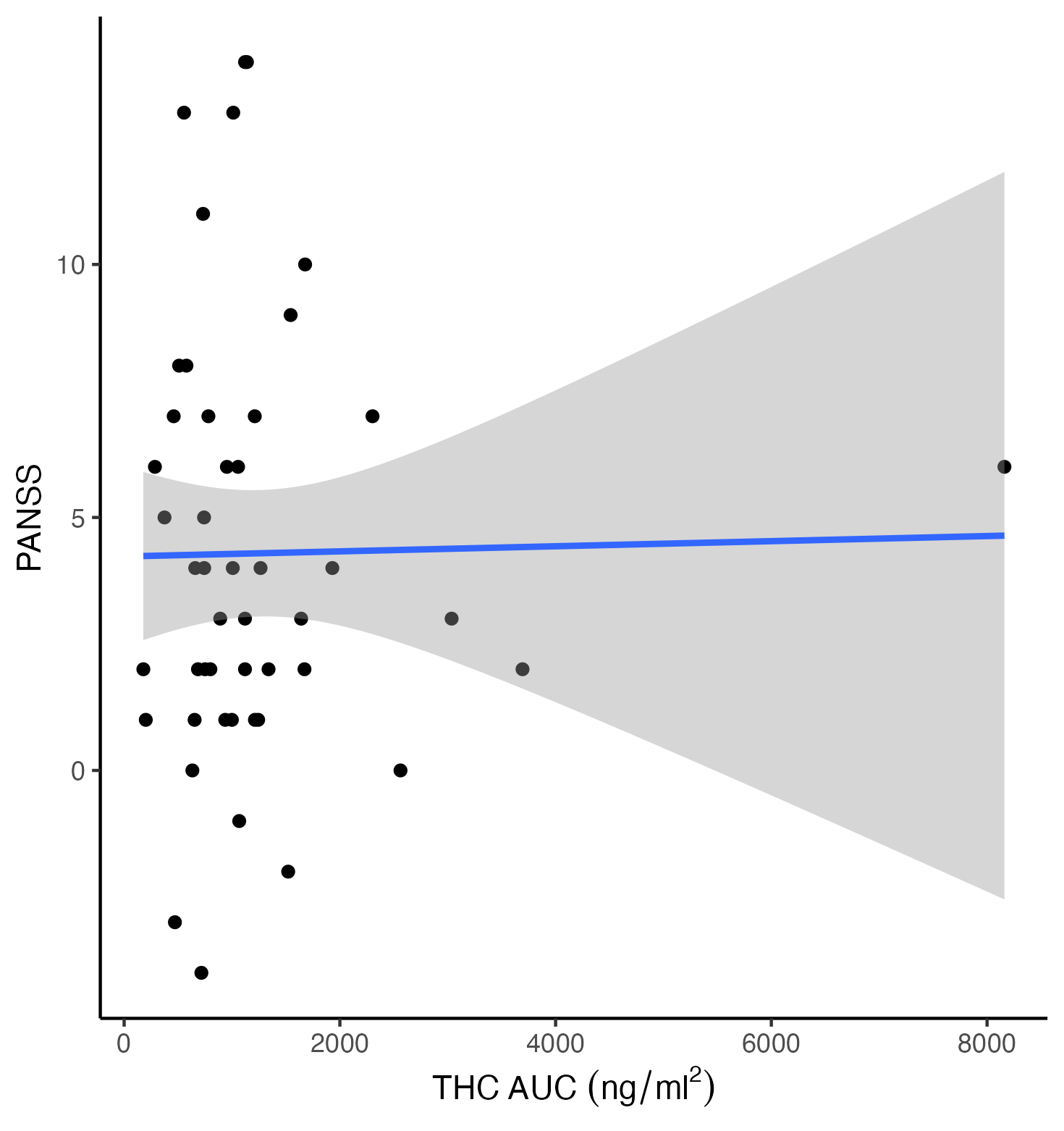

Supplement: Supplementary file 1 — Supplementary materials [file 41386_2025_2175_MOESM1_ESM.docx]
